# Supplementary material for: Causal Relationship between Aging and Anorexia Nervosa: A White-Matter-Microstructure-Mediated Mendelian Randomization Analysis
Source: Biomedicines. 2024 Aug 16;12(8):1874. doi: 10.3390/biomedicines12081874 (PMC11351342; doi:10.3390/biomedicines12081874)
Supplement: Supplementary file 1 [file biomedicines-12-01874-s001.zip › Supplementary Figure/Figure S8/Leaveoneout plot of MR analysis for TL to ubm-b-1490.pdf]

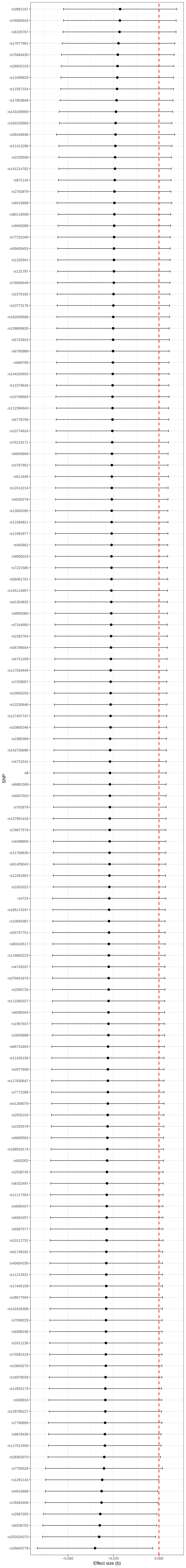
SNP
Effect size (b)
-0.050
-0.025
0.000
rs3891167
rs76065543
rs8105767
rs17677991
rs75664430
rs28502153
rs11699829
rs11557154
rs17803849
rs143190905
rs150150565
rs35446936
rs11412296
rs2230590
rs141214782
rs871134
rs2763979
rs9419958
rs80116508
rs9940099
rs77231040
rs59409453
rs1332941
rs131797
rs76666449
rs2276182
rs10773176
rs182059586
rs139669835
rs5742915
rs6790988
rs869785
rs144204502
rs11579626
rs10768683
rs112394943
rs6776756
rs10774624
rs76219171
rs6659669
rs3767952
rs611646
rs12412214
rs4530278
rs13062095
rs11584821
rs11991877
rs450962
rs9600019
rs7221585
rs56061761
rs145114957
rs41304832
rs9955360
rs7164950
rs2282764
rs56799554
rs6751209
rs117034449
rs7209057
rs10905255
rs13230646
rs117407747
rs10805346
rs1985369
rs142730696
rs4731541
A8
rs6881568
rs6007020
rs762679
rs137901416
rs79977579
rs4498805
rs11769630
rs61405042
rs12451892
rs1003322
rs4724
rs185174247
rs10845387
rs55747751
rs80324517
rs116863223
rs4743037
rs376641875
rs2056726
rs111950327
rs6590343
rs1957927
rs3093888
rs66731853
rs11426156
rs2977608
rs117630647
rs7772289
rs41269079
rs2555104
rs2293579
rs6669563
rs188918174
rs932002
rs2538745
rs8102497
rs11117354
rs4695407
rs6054257
rs6587577
rs10112752
rs61748181
rs45604339
rs12212631
rs17445108
rs28577594
rs142426306
rs7099229
rs9398196
rs1611236
rs73581419
rs10840270
rs16978028
rs12932179
rs939916
rs139795227
rs7790856
rs9878436
rs117512405
rs28363070
rs7705526
rs1291143
rs4616688
rs78491606
rs2967355
rs6536702
rs202034370
rs35640778
